# Evidence for Transcriptional Activity in the Syncytiotrophoblast of the Human Placenta

**DOI:** 10.1016/j.placenta.2009.01.002

**Published:** 2009-04

**Authors:** P.M. Ellery, T. Cindrova-Davies, E. Jauniaux, A.C. Ferguson-Smith, G.J. Burton

**Affiliations:** a Centre for Trophoblast Research, University of Cambridge, Downing Street, Cambridge CB2 3EG, UK; b Department of Physiology, Development and Neuroscience, University of Cambridge, Downing Street, Cambridge CB2 3EG, UK; c Academic Department of Obstetrics and Gynaecology, Royal Free and University College, London, UK

**Keywords:** Placenta, Syncytiotrophoblast, Syncytial knots, Transcription, RNA polymerase II

## Abstract

The aim was to test for evidence of transcriptional activity within the nuclei of the syncytiotrophoblast of the human placenta. The syncytiotrophoblast forms the epithelial covering of the villous tree, and is a multinucleated, terminally-differentiated syncytium generated through fusion of the underlying progenitor cytotrophoblast cells. Its nuclei are heterogeneous with respect to chromatin condensation, and previous functional studies of 3H-uridine uptake *in vitro* have indicated that they are transcriptionally inactive. This observation is surprising given the key roles this tissue plays in active transport, hormone synthesis and metabolic regulation, and has widespread implications for trophoblast physiology and pathophysiology. We used three different approaches to look for evidence of transcriptional activity. First, immunofluorescence staining was performed on paraffin-embedded early pregnancy and term placental villi, using an antibody directed specifically against the actively transcribing form of RNA polymerase II. Second, a nucleoside incorporation assay was applied to placental villi maintained in short-term culture, with and without the transcription blocker α-amanitin. Third, histone modifications associated with active chromatin were identified by immunohistochemistry and immunofluorescence. Each of these methods showed transcription to be occurring in a proportion of syncytiotrophoblast nuclei, with qualitative evidence for transcription being more abundant in the first trimester than at term. These findings correlated with electron microscopical observations of prominent nucleoli within the nuclei, particularly during early pregnancy, signifying transcription of ribosomal RNA. Contrary to previous findings, these results confirm that a proportion of syncytiotrophoblast nuclei actively produce mRNA transcripts.

## 1 Introduction

Trophoblast forms the epithelial covering of the human placental villous tree, and is the interface with the maternal blood. Villous trophoblast consists of two cell populations, the cytotrophoblast (CTB) cells and the syncytiotrophoblast (STB). The STB is a unique tissue, for it is an extensive terminally-differentiated multinucleated syncytium with a surface area of approximately 12 m^2^ at term. Mitotic figures have never been observed within the STB, but instead it is generated through proliferation, differentiation and subsequent fusion of CTB cells [1]. Nuclei within the STB will therefore be of different ages depending on the time of their incorporation, and this may explain the range in their morphological appearances [2]. Whilst some nuclei display an open euchromatic pattern, often with a prominent nucleolus, others consist almost entirely of dense heterochromatin. The latter are considered to be aged, effete nuclei, and are usually sequestered together to form aggregates referred to as syncytial knots [3]. These are rarely seen in normal placentas prior to 32 weeks of pregnancy, become more frequent towards term, and are a conspicuous feature of the post-mature placenta [4]. In pathological placentas there is a modest increase in syncytial knots associated with preeclampsia, but they are more strongly associated with disorders of fetal perfusion of the villous tree, such as stem artery thrombosis [4].

Because of these differences in chromatin condensation it might be expected that STB nuclei show a range of transcriptional activity. However, results from a functional assay involving the incubation of both first trimester and term placental villi with a radiolabelled nucleoside, 3H-uridine, revealed no incorporation into the STB nuclei, suggesting a global suppression of transcriptional activity [5,6]. The authors concluded that there is a continual need for CTB fusion to bring fresh transcripts into the STB, and proposed a transit of nuclei through the STB based around apoptosis [7,8]. This lack of transcription is surprising, given the high secretory and metabolic activities of the STB. It would also appear to hamper the responsiveness of the tissue to sudden changes in the intrauterine environment, for the requirement for cell fusion to introduce more, or different, transcripts must introduce an inevitable delay.

We therefore tested for evidence of transcriptional activity in the STB nuclei at various gestational ages using three different techniques. Firstly, immunofluorescent staining for the actively transcribing form of RNA polymerase II was performed on both early pregnancy and term placental samples. Secondly, a nucleoside incorporation assay using fluorouridine was performed on placental explants maintained under physiological conditions *in vitro*. Thirdly, immunohistochemistry was performed for histone methylation patterns associated with active transcription. In addition, transmission electron microscopy was performed to examine nuclear morphology.

## 2 Materials and methods

### 2.1 Sample collection and tissue processing

Tissue collection was approved by the relevant local research ethics committees in Cambridge and London. Human placentas were obtained, with informed consent, from normal, uncomplicated pregnancies undergoing either surgical termination at 5–17 weeks gestational age or elective caesarean delivery at term. Samples were fixed in 4% paraformaldehyde (PFA) overnight at 4 °C for immunohistochemistry, or in 2% glutaraldehyde for 2 h for electron microscopy.

### 2.2 Immunofluorescence and immunohistochemistry

For immunofluorescence, PFA-fixed tissues were embedded in paraffin wax, sectioned at 7 μm, and dewaxed. Antigen retrieval was then performed using 0.02 mg/ml proteinase K (Sigma, Poole, UK) for 30 min at room temperature. Sections were then incubated with non-immune serum for 20 min. A mixture of primary antibodies, including anti-RNAP II (Covance, Emeryville, CA, USA), anti-cytokeratin 7 (DAKO, Ely, UK), anti-H3K4Me2 (Millipore, Consett, UK), and anti-H3K4Me3 (Abcam, Cambridge, UK), was applied overnight at 4 °C. The sections were washed and incubated for 1 h at room temperature with species-specific Alexa-488 or Alexa-568 secondary fluorescent antibodies (Molecular Probes, Invitrogen Detection Technologies, Leiden, The Netherlands). Sections were washed in TBS and finally in water, and subsequently mounted in Vectashield mounting medium containing DAPI (Vector Laboratories, Peterborough, UK). Images were captured using a Leica confocal microscope (LeicaTCS-NT, Leica Instruments GmbH, Germany).

The above protocol was also followed for immunohistochemistry, with the following exceptions. Endogenous peroxidase activity was blocked prior to primary antibody treatment by incubation in 3% H_2_O_2_ for 15 min at room temperature. Biotin-labelled secondary antibodies were used and binding was detected using Vectastain Elite ABC kits (Vector Laboratories, Peterborough, UK) and SigmaFast DAB (Sigma, Poole, UK), according to the manufacturers' instructions. Sections were then lightly counterstained with haematoxylin. Negative controls were performed by replacement with an equal concentration of non-immune or isotype matched irrelevant control.

### 2.3 Fluorouridine (FU) incorporation assay

The following protocol was developed from the work of Dimitrova and of Boisvert et al. who applied the technique to assay transcription in cell culture work, and from the methods of Watson et al. who studied incorporation of bromodeoxyuridine (BrdU) into placental villi [9–11].

Villi from first trimester and term placentas were placed immediately into culture medium (TCS large vessel endothelial cell basal medium (TCS CellWorks, Milton Keynes, UK), containing 2% fetal bovine serum, heparin, hydrocortisone, human epidermal growth factor, human basic fibroblast growth factor, 25 μg/ml gentamicin and 50 ng/ml amphotericin B, 1 mM vitamin C and 1 mM Trolox) pre-equilibrated with 2.5% O_2_/92.5% N_2_/5% CO_2_. FU was added to the experimental samples to a final concentration of 2 mM. Control samples were placed in medium not containing FU. Samples were then incubated at 37 °C for 1 h. For term placentas, control samples were pre-treated with an RNAP II inhibitor, α-amanitin (40 μg/ml; Sigma), for 30 min at 37 °C, followed by incubation with 2 mM FU for a further 1 h. Fixation and paraffin-embedding were carried out on all samples, as described above.

FU was detected immunohistochemically with an anti-BrdU mouse monoclonal antibody (Sigma) which reacts with both BrdU and FU [10]. Immunohistochemistry was carried out as described above, with several modifications. A 30 min incubation with 2 M HCl at 37 °C was performed prior to anti-BrdU treatment to cause DNA denaturation. Instead of proteinase K, slides were incubated individually with trypsin solution (Sigma) for 20 min at 37 °C. Slides were not counterstained with haematoxylin; instead, they were rinsed in dH_2_O and cover-slipped with a permanent aqueous mount.

### 2.4 MTT assay

To confirm syncytial viability, an MTT test was performed, as previously described [11]. Ten to fifteen milligram samples of placental villi were placed in culture medium containing 0.5 mg/ml 3-(4,5-dimethyldiazol-2-yl)-2,5-diphenyltetrazolium bromide (MTT), and incubated for 20 min at 37 °C in the dark. Sections were frozen in OCT (Tissue-Tek), then sectioned on a cryotome and mounted onto microscope slides. Mitochondrial activity was demonstrated by the incorporation of a blue formazan cleavage product.

### 2.5 Electron microscopy

Following secondary fixation in 1% osmium tetroxide for 1 h, samples were embedded in Araldite epoxy resin. Semi-thin sections (1 μm) were stained with methylene blue, whereas ultra-thin sections (50 nm) were counterstained with uranyl acetate followed by lead citrate and viewed using a Philips CM100 microscope.

## 3 Results

### 3.1 RNA polymerase II (RNAP II) immunofluorescence

**Fig. 1:**
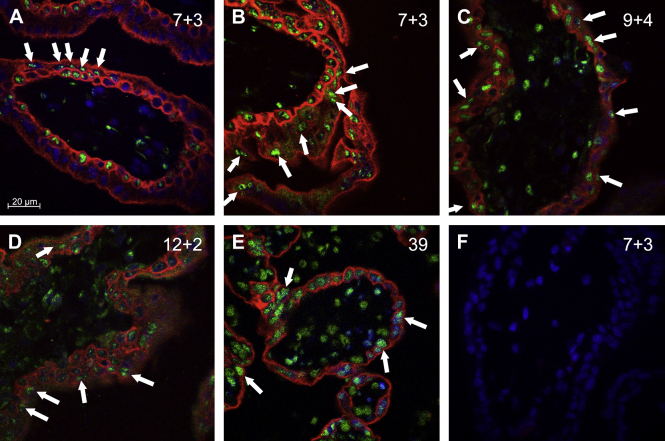
Detection of active RNAP II. (A–E) Immunofluorescence for RNAP II (green), cytokeratin 7 (red) and DAPI (blue). Examples of RNAP II-immunoreactive STB nuclei are illustrated by arrows. (F) Negative control (omission of primary antibodies). Gestational ages (weeks + days) are given at the top right of the images. Scale bar = 20 μm for all images.

Immunofluorescence staining for RNAP II and cytokeratin 7 was carried out on villi from eight early pregnancy placentas, ranging from 5 to 14 weeks in age, and at term (Fig. 1A–E). Cytokeratin 7 was used to identify trophoblast cell boundaries, and thus to discriminate between the STB and CTB. Immunoreactivity for RNAP II was seen in a proportion of the CTB, STB and stromal nuclei in all the samples. The proportion of nuclei staining in these tissues varied with gestational age. Early in the first trimester, 5–8 weeks (*n* = 4), the villous trophoblast was variable in its immunoreactivity, with some regions almost devoid of RNAP II-positive nuclei, and others abundant in them (Fig. 1A and B). Immunoreactivity of the STB seemed to correlate with the presence of RNAP II in the underlying CTB, but not *vice versa*. Stromal staining for RNAP II was infrequent.

Later in the first trimester and early second trimester (9–14 weeks, *n* = 4), RNAP II-immunoreactive nuclei were more abundant, and were seen in the trophoblast and stroma of almost all villi (Fig. 1C and D). A similar pattern was observed at term (*n* = 4) (Fig. 1E). Autofluorescence and non-specific binding of the secondary antibody were negligible, as demonstrated by the lack of staining in the negative controls (Fig. 1F).

On the subnuclear level, RNAP II was variable in quantity, ranging from almost complete coverage of the nucleus to just small clumps of staining. Even in the most densely staining nuclei, RNAP II staining was seen to be absent from the central region of the nucleus, presumed to be the nucleolus, providing evidence of selectivity for this form of the polymerase.

### 3.2 FU incorporation assay

**Fig. 2:**
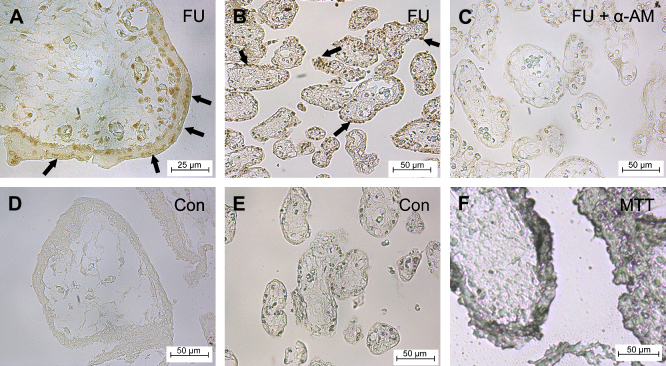
FU incorporation into placental tissue, detected by immunohistochemical staining. Brown staining on the light photomicrographs indicates the presence of FU, in: (A) first trimester (9 + 2 week) placenta; (B) term caesarean placenta; (C) term caesarean placenta incubated with α-amanitin; (D) first trimester control placenta. (E) Term caesarean control placenta. STB viability was confirmed by the presence of blue staining in an MTT test, as shown in (F) for first trimester placenta. Arrows indicate immunoreactive STB nuclei. Scale bar = 25 μm for (A), and 50 μm for (B–F).

Villi from first trimester placentas (*n* = 2) that were incubated with FU and stained with an anti-BrdU antibody displayed widespread immunoreactivity of trophoblast nuclei. Immunoreactivity was seen in both CTB and STB nuclei, the latter being identified by their proximity to the outer surface of the villus (Fig. 2A, arrows). The staining intensity of the STB appeared to be greater than that of the CTB in many cases. Control villi obtained from the same placentas and incubated in the culture medium without FU displayed no evidence of immunoreactivity in the trophoblast or stroma when treated with the same immunohistochemistry protocol (Fig. 2D, E).

In term placentas (*n* = 3), the abundance of densely staining nuclei was much lower and was confined to the periphery of the villi studied (Fig. 2B), but was still consistently observed, with controls showing no immunoreactivity for FU (Fig. 2E). In villi incubated with both α-amanitin and FU, staining was virtually absent (Fig. 2C).

### 3.3 MTT assay

Blue staining was seen in the trophoblast of both early pregnancy and term villi (Fig. 2F), demonstrating that mitochondrial function was preserved during the incubation period. The cryostat sections showed a thick trophoblast layer with no evident detachment from the underlying stroma, suggesting that no syncytial degeneration had occurred (Fig. 2F).

### 3.4 Immunostaining for histone modifications

**Fig. 3:**
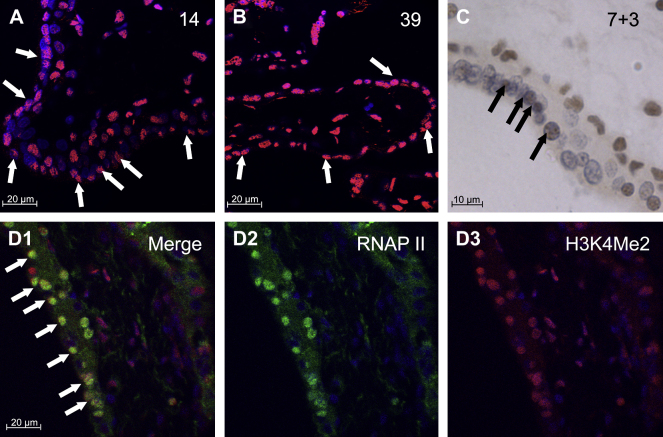
Detection of modified histones. (A and B) Immunofluorescence for H3K4Me2 (red) and DAPI (blue). Arrows indicate immunoreactive STB nuclei. (C) Immunohistochemistry for H3K4Me3 (brown). Nuclei are counterstained with haematoxylin (blue). (D1–3) Immunofluorescence for H3K4Me2 (red), RNAP II (green) and DAPI (blue) (gestational age 9 + 3 weeks). Arrows indicate co-localisation of H3K4Me2 and RNAP II immunoreactivity in STB nuclei. Arrows indicate immunoreactive STB nuclei. Gestational ages (weeks + days) are given at the top right of the images. Scale bar = 20 μm for (A), (B) and (D), and 10 μm for (C).

Immunofluorescent staining of first trimester (*n* = 6) and term (*n* = 4) placental villi for dimethylated lysine 4 on histone H3 (H3K4Me2) demonstrated widespread nuclear immunoreactivity in both layers of the trophoblast and in the stroma (Fig. 3A and B). Additionally, H3K4Me2 often co-localised with active RNAP II in the trophoblast and stromal nuclei of all the first trimester villi examined (*n* = 4) (Fig. 3D1–3). Some nuclei were observed to contain the modified histone but not RNAP II, but not *vice versa*. Autofluorescence and non-specific binding were negligible, as demonstrated by a lack of staining in the negative controls (not shown).

Immunohistochemistry was also performed on first trimester (*n* = 5) placental villi for dimethylated lysine 4 on histone H3 (H3K4Me3). Highly selective nuclear immunoreactivity was seen, and was mainly localised to stromal and CTB cells, with little involvement of the STB (Fig. 3C). A negative control showed non-specific binding of the secondary antibody to be negligible (not shown).

### 3.5 Electron microscopy

**Fig. 4:**
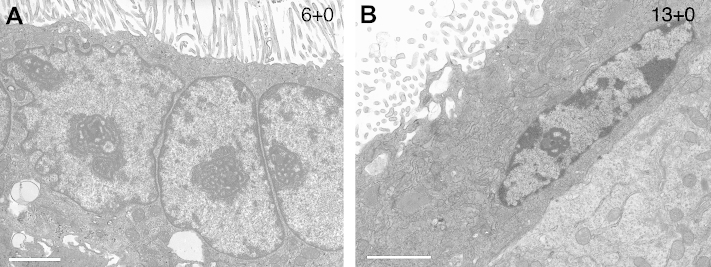
Electron micrographs showing examples of euchromatic STB nuclei with prominent nucleoli. Gestational ages (weeks) are given at the top right of the images. Scale bar = 2 μm.

In the earliest gestational age samples examined the STB nuclei displayed an open euchromatic appearance similar to that in the CTB cells, with only occasional small aggregates of heterochromatin at the margins or within the nuclear profile. Nucleoli were a prominent feature in nearly all these nuclei (Fig. 4A). As gestation advanced into the second trimester heterochromatin became more conspicuous, but still occupied only a relatively small percentage of the nuclear profiles. Nucleoli were still seen, but less frequently (Fig. 4B). These trends were more exaggerated at term, when dense aggregates of heterochromatin were present in some STB nuclei, and nucleoli were rare (data not shown).

## 4 Discussion

### 4.1 Evidence for STB transcription

The results of all three experimental approaches confirmed that active transcription occurs in a sub-set of the STB nuclei. Qualitatively, it appears that such nuclei are more frequent in early second trimester tissue than at term, but further quantitative studies are required to confirm this impression.

RNAP II is the polymerase responsible for the synthesis of mRNA (as opposed to rRNA or tRNA), and the antibody we used to detect it was specific to H5, a serine-phosphorylated form of the polymerase only found in actively transcribing cells [12,13]. We can thus infer that the observed immunoreactive STB cells are producing nascent mRNA transcripts. The observation that RNAP II-positive STB nuclei tend to cluster in regions where CTB immunoreactivity is also observed may be indicative that the active STB nuclei are those that have recently been incorporated into the tissue. This interpretation is consistent with the euchromatic appearance of fully differentiated CTB cells prior to fusion with the STB [14].

The FU incorporation assay provided further evidence that transcription is occurring in both the trophoblast and stromal cells. No immunoreactivity was seen in the negative controls where FU was omitted, or following pre-treatment and incubation with α-amanitin. This potent and selective RNAP II inhibitor blocks the elongation of nascent transcripts [15,16], and has previously been used successfully on placental explants [17]. The only finding from the FU incorporation experiment inconsistent with the RNAP II results was that STB nuclei were often more strongly immunoreactive than CTB or stromal cells. The most likely explanation for this effect was that there was insufficient time during the incubation period for the FU to diffuse towards nuclei deeper in the villous core. The incubation period was kept short to ensure viability of the STB throughout the experiment [18], and this was confirmed by the MTT assay.

Further information concerning the transcriptional status was obtained by looking for the presence of histone modifications. Histones are highly conserved, basic proteins that package the DNA into chromatin and influence genome architecture and function. They can undergo enzyme-catalysed covalent modifications, which can affect their interactions with DNA, many leading to the recruitment of further proteins with diverse consequences [19]. The modifications we examined were di- and trimethylated H3K4 (H3K4Me2 and H3K4Me3, respectively) that are associated with active euchromatic regions in mammalian cells, with H3K4Me3 in particular being enriched at transcriptionally active promoters [20–22]. Our results show both modifications to be present in a proportion of villous trophoblast cells, and the observation that a marker for active chromatin, H3K4Me2, co-localised with active RNAP II in many STB nuclei provides further strong evidence that transcription is taking place.

Despite this finding, the distribution of H3K4Me3 was less extensive in the STB than might be expected from our RNAP II results. An explanation may be provided by the work of Kimura and colleagues, who used chromatin immunoprecipitation to examine histone modifications at the human growth hormone (*hGH*) gene cluster in fusing primary CTBs isolated from human term placentas [23,24]. Activation of these genes during fusion is associated with robust acetylation of histones H3 and H4, but the lower quantity and temporal pattern of H3 lysine 4 methylation suggest it plays a role in ‘priming’ the genes for activation [24].

These data are consistent with ultrastructural observations of the STB nuclei. The majority of the nuclei in the first trimester, but also a small proportion at term, display a prominent nucleolus, consistent with previous observations [2]. The nucleolus is the site of rRNA production [25], indicating that the STB has the capacity to produce its own ribosomes rather than being passively replenished when CTB cells fuse into the syncytium. It would be rather unusual for a nucleus to be able to transcribe one type of RNA but not another.

### 4.2 Comparison with 3H-uridine labelling data

As mentioned earlier, previous *in vitro* experiments revealed little or no incorporation of 3H-uridine into either first trimester or term STB nuclei [5,6]. This disparity with the current findings may reflect the contrasting culture conditions employed. In the previous studies villous explants were maintained for 1 h under 95% oxygen, 5% carbon dioxide at 2.5 atmosphere pressure, conditions which are now recognised as being hyperoxic for placental tissues *in vivo* [26]. Although viability of the STB was maintained throughout the culture period, it is possible that an as yet unidentified stress led to the suppression of transcription under these conditions.

### 4.3 Implications of STB transcription

The ability of the STB to produce its own mRNA transcripts is in keeping with its high metabolic and secretory activity. For example, towards term the STB secretes approximately 1 g of placental lactogen *per* day [27], and the transcripts encoding this hormone account for approximately 10% of all placental transcripts. *In situ* hybridization using tritiated probes has localised these transcripts exclusively to the STB in first trimester samples, with only a few scattered grains overlying the CTB cells [28].

It will also permit the STB to adapt rapidly to changes in the intrauterine environment without any delay caused by the additional need for CTB fusion. It was notable that on all the immunofluorescence slides examined, villi from 9 to 14 weeks gestation displayed more widespread RNAP II immunoreactivity in all tissue compartments than villi from 5 to 8 weeks. Furthermore, their nuclei tended to display a greater density of RNAP II staining, a variable associated with the number of nascent transcripts being produced [29]. This difference may relate to the onset of the haemochorial circulation to the placenta, when there is a threefold increase in the oxygen concentration in the maternal intervillous space. The change in transcriptional activity may be associated with the rise in mRNA transcripts encoding antioxidant enzymes in villous tissue at that time [26], and/or may reflect a general increase in the metabolic activity of the tissue driven by the higher oxygen availability.

Not all STB nuclei appear to be transcriptionally active, however, indicating that some form of selective regulation does occur. Quantitative data obtained using the disector technique reveal that the number of STB nuclei increases exponentially until term, increasing from 6.2 × 10^9^ at 13–15 weeks to 58.1 × 10^9^ at 37–39 weeks [30]. Whether only a proportion of these is needed to produce sufficient transcripts, given the syncytial nature of the tissue, or whether nuclei that have sustained oxidative or some other form of damage are preferentially inactivated is not known.

Alternatively, the different transcriptional states of these cells may reflect distinct sub-populations with different properties. Further quantitative studies are required to determine whether the proportion of active nuclei remains constant across pregnancy and in pathological conditions, for qualitative impressions based on viewing sections are potentially misleading. Spatial dispersal of the RNAP II-positive nuclei as the villous tree enlarges may create a false impression as to their true frequency, as previously demonstrated in the case of CTB cells [31].

The mechanism by which STB transcription is controlled is also unclear. That there is heterogeneity in the chromatin pattern amongst STB nuclei, and that the frequency of syncytial knots increases towards term, are incontrovertible [3,32]. It has been suggested that these changes represent progression along the apoptotic pathway [8]. Indeed, it was proposed that activation of the apoptotic cascade is a key event during CTB fusion, but that once the nucleus is incorporated into the STB, progression is delayed for several weeks due to high levels of Bcl-2 carried in from the CTB cytoplasm [7]. Cytotrophoblast cells can, and do, undergo apoptosis [14], but whether apoptosis is part of the fusion mechanism has recently been questioned [33,34]. Nuclear blebbing and fragmentation are not observed in syncytial knots; indeed Jones and Fox commented on the very smooth contours of the nuclei, permitting their close juxtaposition [32]. Another possibility is that histone modifications, such as methylation, phosphorylation or acetylation, may lead to repackaging and inactivation of the DNA, and further work is required to explore this possibility.

In conclusion, we have provided direct and indirect evidence that a proportion of nuclei in the syncytiotrophoblast of the human placenta is actively engaged in producing RNA transcripts, both in early pregnancy and at term. Future experimental work is seeking to quantify the proportion of nuclei that are transcriptionally active, and examine the molecular mechanisms by which activity is regulated.
